# Pharmacological investigations of effort-based decision-making in humans: Naltrexone and nicotine

**DOI:** 10.1371/journal.pone.0275027

**Published:** 2022-10-05

**Authors:** Cecilia Nunez, Jennifer K. Hoots, Scott T. Schepers, Michael Bower, Harriet de Wit, Margaret C. Wardle

**Affiliations:** 1 Department of Psychology, University of Illinois Chicago, Chicago, Illinois, United States of America; 2 Department of Psychiatry and Behavioral Neuroscience, University of Chicago, Chicago, Illinois, United States of America; Bangor University, UNITED KINGDOM

## Abstract

Many mental health disorders are characterized by an impaired ability, or willingness, to exert effort to obtain rewards. This impairment is modeled in effort-based decision tasks, and neuropharmacological studies implicate dopamine in this process. However, other transmitter systems such as opioidergic and cholinergic systems have received less attention. Here, in two separate studies we tested the acute effects of naltrexone and nicotine on effort-based decision-making in healthy adults. In Study 1, we compared naltrexone (50mg and 25mg) to placebo, and in Study 2, a pilot study, we compared nicotine (7mg) to placebo. In both studies, participants completed the Effort Expenditure for Rewards Task (EEfRT), which measured effort-based decision-making related to monetary rewards. Although subjects expended greater effort for larger reward magnitude and when there was a higher probability of receiving the reward, neither naltrexone nor nicotine affected willingness to exert effort for monetary rewards. Although the drugs produced significant and typical drug effects on measures of mood and behavior, they did not alter effort-based decision-making. This has implications both for the clinical use of these drugs, as well as for understanding the neuropharmacology of effort-related behavior.

## Introduction

Effort-based decision-making, or choosing when to exert effort to gain rewards, is important for effective behavioral functioning, and is impaired in many psychopathologies including substance use disorders, schizophrenia, posttraumatic stress disorder, and depression [1–5]. Thus, it is important to understand how both abused and medicinal drugs may affect effort-based decision-making in humans. Preclinical studies show effort-based decision-making is most prominently regulated by dopamine [6], and consistent with this, dopaminergic drugs alter effort-based decision-making in humans [7–10]. However, preclinical studies show that opioidergic and cholinergic systems also play a significant role in effort-based decision-making [11, 12]. However, less is known about how opioidergic and cholinergic drugs affect effort-based decision-making in humans.

Opioidergic manipulations might be expected to affect effort because opioidergic “hedonic hotspots” in the nucleus accumbens appear to regulate pleasure or "liking" for rewards [13–16]. Opioidergic agonists, which increase "liking" of reward, might thus increase effort to obtain rewards, while antagonists would be expected to decrease effort. This might be particularly evident over repeated trials as organisms experience altered hedonic “payoffs” for their effort. Consistent with this theory, opioid agonists in rats increase the value of rewards and the effort exerted to obtain rewards [11, 17, 18], while antagonists, such as naltrexone, reduce liking and consumption of rewards [13] as well as willingness to exert effort for rewards [19–22]. The literature in humans is more limited. Studies in healthy humans show that acute administration of the μ-opioid receptor antagonists naloxone and naltrexone reduce preference for high-value food rewards [15, 23] and reduce both self-reported pleasure and activity in the anterior cingulate cortex (an area associated with valuation) to rewarding gambling outcomes [24]. These findings are consistent with reduced pleasure, but do not directly address effort. In healthy humans, acute naltrexone also reduces two indirect measures of effort: it slows reward learning in a decision-making task, which the authors attribute to decreased effort exerted to attend to reward cues [15, 25], and it decreases attention to the information-rich eye region of photos, suggestive of reduced motivation for valuable social cues [26]. Finally, acute naltrexone administration disrupts computations of reward value in a Pavlovian instrumental transfer task in healthy humans [27]. However, all these studies used indirect measures of effort, rather than directly studying effort/reward tradeoffs. Thus, additional research is needed to understand whether opioidergic manipulations directly affect effort-based decision making in humans.

Drugs affecting the nicotinic acetylcholine system would also be expected to affect effort. Here we base our predictions on the "dual reinforcement model" of nicotine addiction. This model proposes that nicotine both serves as a primary reinforcer *and* enhances the rewarding properties of concurrent non-drug rewards, in part through interactions with the mesolimbic dopamine systems [28, 29]. If nicotine increases the rewarding properties of non-drug rewards, we would expect nicotine to enhance effort for these rewards as well. However, here the rodent literature presents mixed findings. Some studies find that nicotine increases operant responding with visual rewards [30], whereas others find nicotine either has no effect or even decreases effort for food rewards [31]. In humans, the question of whether nicotine enhances effort for rewards has often been studied in dependent smokers [32–34], but these results are complicated by receptor adaptations, tolerance and the reward-blunting effects of nicotine withdrawal. However, there is evidence that in non-nicotine-dependent individuals acute nicotine administration increases responding for sensory rewards (i.e., audio, olfactory, taste, visual stimuli), but not for non-sensory rewards (i.e., money) [29, 35]. The neuropharmacological mechanism behind this sensory/non-sensory distinction is unclear, and the question of whether nicotine specifically affects effort to obtain rewards has not been addressed using a standardized effort-based decision-making task.

Here, we tested the acute effects of naltrexone and nicotine on effort expenditure in two separate studies. We used the Effort Expenditure for Rewards Task (EEfRT), which has previously been sensitive to dopamine manipulations in humans [7, 36, 37]. In Study 1, we compared naltrexone (25mg and 50mg) to placebo. We hypothesized naltrexone would reduce willingness to exert effort for rewards. We also hypothesized this effect would be more evident in later trials, after participants had repeatedly experienced lower pleasure from rewards. In Study 2, a pilot study, we compared nicotine (7mg) to placebo. We hypothesized nicotine would increase willingness to exert effort for rewards. We also hypothesized nicotine would have a greater effect on willingness to exert effort for rewards in trials with lower reward probabilities, because, in previous studies, willingness to exert effort has been particularly pronounced for trials when reward probability was lower [7, 37].

## Materials and methods

### Manipulation checks

We assessed mood and physiological effects of the drugs to confirm that they produced their expected effects. In both studies, we measured subjective effects of naltrexone and nicotine with the Drug Effects Questionnaire (DEQ) [38], which contains items asking the extent to which participants felt the drug, liked the drug, and disliked the drug. We assessed cardiovascular effects on heart rate and blood pressure (diastolic and systolic) [39]. In Study 1, we also measured subjective effects with the Profile of Mood States (POMS) [40]. Naltrexone manipulation checks were conducted at baseline, 30 min. post-administration, 75 min. post-administration, 180 min. post-administration, and 210 min. post-administration. Nicotine manipulation checks were measured at baseline, 60 min. post-administration, 100 min. post-administration, and 160 min. post-administration.

### Effort Expenditure for Rewards Task (EEfRT)

The EEfRT measures effort-based decision-making [36] via a computerized game consisting of 50 trials. In each trial, participants are prompted to choose either an easy task or a hard task to earn monetary rewards of varying amounts. During the choice period, individuals are presented with the probability of the trial being a “win” trial (12, 50, or 88%) and the amount of money they could earn for the hard task ($1.24-$4.20). Easy tasks are always worth $1. Participants can earn money if they successfully complete the trial, and the trial is a “win” trial. “No win” trials result in $0. Participants then choose which task they will attempt and begin the task. To successfully complete the easy task, participants must press a key with their dominant index finger 30 times in 7 seconds. To successfully complete the hard task, participants must press a key with their non-dominant hand’s little finger 100 times in 21 seconds. After the attempt is complete, participants receive feedback informing them how much money they won during the trial. At the end of the task, participants received monetary rewards from two, randomly selected, “win” trials that they successfully completed. The primary outcome variable modeled is whether the participants selected the high cost/high reward option over the low cost/low reward option on a given trial.

For both studies, subjects were excluded if they failed to complete 40 trials in one session, if they always chose the easy or hard task, only completed “high value” trials (this is a strategy that can artificially increase payouts on this task) or if they did not consistently choose the hard task more as probability of reward increased (indicating non-systematic responding). This exclusion criteria is similar to that of previous studies which used the EEfRT [37]. One participant was excluded in Study 1, and 3 participants were excluded in Study 2.

### Study 1 naltrexone

#### Overall design

These data were obtained as part of a larger study that was examining the effect of naltrexone on processing of social and emotional stimuli [41]. The study used a three-session, within-subjects design in which healthy volunteers received oral placebo, 25mg or 50mg naltrexone in a counterbalanced order under double-blind conditions. Sessions were separated by at least 1 week, and the EEfRT was completed 90 minutes after drug administration, during the expected peak effect for naltrexone. During the interval between administration and EEfRT, subjects were permitted to relax and watch a movie or read a book, but they were not allowed to engage in work. The EEfRT was completed as part of a 1.5hr battery of social/emotional tasks, which were administered in randomized order. Results of the other tasks are reported elsewhere [41]. Subjects completed rating scales and cardiovascular measures were obtained at regular intervals during the session.

#### Participants

Thirty-three healthy adults (18–35 years old) participated (Table 1). They were screened with a physical examination, electrocardiogram, modified structured clinical interview for the Diagnostic and Statistical Manual of Mental Disorders, 4th Edition (DSM-IV) [42], and self-reported drug and health history. Criteria for inclusion were a high school education with English fluency and body mass index (BMI) of 19–30. Exclusion criteria included regular use of prescription medications (except oral contraceptives), medical contraindication to naltrexone, prior negative reactions to naltrexone, regular use of opioid drugs, past-year DSM-IV Axis I diagnosis (except Nicotine Dependence), and women who were pregnant or planning to become pregnant. Participants abstained from alcohol and medications (except hormonal contraceptives) for 24 hours before sessions and abstained from other drugs (except caffeine and nicotine) for 48 hours before sessions. They were instructed to fast for 2 hours. Compliance was verified using self-report, plus breath (Alcosensor III, Intoximeters Inc., St. Louis, MO) and urine tests (ToxCup, Branan Medical Corporation, Irvine, CA) for alcohol and drugs. Women not on hormonal contraceptives were scheduled during the follicular phase of their menstrual cycle [43]. To reduce expectancy effects, individuals were told they might receive a stimulant, a sedative, a cannabinoid, an opioid antagonist, or a placebo.

**Table 1:** Sample characteristics for Study 1 (Naltrexone) and Study 2 (Nicotine).

*Demographics*	Naltrexone	Nicotine
	*M (SD) / % (n = 33)*	*M (SD) / % (n = 15)*
Age	24 (3.62)	23.93 (4.17)
Gender (% Female)	47.06%	46.67%
Education (years)	15.24 (1.61)	15.07 (1.49)
Race		
Asian	11.76%	6.67%
Black/African American	23.53%	0%
White	50%	66.67%
Other	14.71%	26.67%
Ethnicity (% Hispanic)	16.13%	20%
BMI	24.63 (2.64)	24.87 (5.49)
*Drug Use History*		
Alcohol (# drinks past 30 days)	28.11(20.42)	40.83(33.26)
Nicotine (# cigarettes past 30 days)	12.14(57.77)	40.5(61.3)

*Note*: For naltrexone, % Hispanic was calculated based on grandparents’ ethnicity.

Participants provided written informed consent, and procedures were conducted in accordance with the Declaration of Helsinki and approved by the University of Chicago Institutional Review Board.

#### Analytic approach

All analyses were mixed models performed in R (Version 3.5.2; [44]) using the lme4 package (Version 1.1–21; [45]). We mean centered all continuous independent variables and contrast coded categorical independent variables. To establish the random effects models, we created a maximal model and iteratively reduced it according to Bates, Kliegl, Vasishth, and Baayen using the RePsychLing package [46, 47]. We used the emmeans package to conduct follow-up tests on significant main effects and interactions (Version 1.5.2–1; [48])

For naltrexone, the “expected effects” used as manipulation checks were subjective fatigue, subjective feeling of a drug effect, and drug disliking. These effects are typical of naltrexone and were all observed in the primary analysis of this data, with only one additional participant excluded here [41]. Subjective (POMS: Fatigue, DEQ: Feel Drug, and DEQ: Dislike Drug) effects were modeled using a linear mixed effects model (LMM), with fixed effects for Drug (0, 25, 50mg), Time (pre-capsule, 30 min. post-capsule, 75 min. post-capsule, 180 min. post-capsule, 210 min. post-capsule), and their interactions. We conducted parallel analyses of heart rate and blood pressure effects for both drugs but did not expect strong effects of naltrexone on these outcomes. These were modeled using a linear mixed effects model (LMM), with fixed effects for Drug, Time (pre-capsule, 30 min. post-capsule, 75 min. post-capsule, 180 min. post-capsule, 210 min. post-capsule), and their interactions. Omnibus tests were conducted on the models using analysis of variance (ANOVA) Type III based on Satterthwaite’s method.

To test the effect of naltrexone on choice in the EEfRT task, we used a generalized linear mixed model (GLMM) with a logit link function for the binomial outcome (choice of easy vs. hard task). Fixed effects were linear and quadratic effects of Naltrexone Dose (0, 25, 50mg), Reward Amount ($1.24-$4.20), linear and quadratic effects of Probability (12, 50, 88%), and their interactions. Fixed effects also included linear and quadratic effects of Session (1, 2, 3), Trial Number (1–50) and its interaction with Naltrexone dose (linear and quadratic), to test the effect of naltrexone on willingness to exert effort across the trials. Omnibus tests were conducted on the model using Type III Wald chi-square tests. Continuous variables of interest were grand-mean centered and converted to Z-scores for all analyses.

### Study 2 nicotine

#### Overall design

To study the effects of nicotine on willingness to exert effort, we conducted a pilot study and used a two-session, within-subjects design. At each session, healthy, light-smoking volunteers received a transdermal patch containing inactive placebo or 7 mg nicotine (Nicoderm CQ, Glaxo Smith Kline) under double-blind conditions. Sessions were separated by at least 24 hours, and the EEfRT was completed 60 minutes after patch administration. At this time, participants’ plasma nicotine concentrations are expected to be approximately 5ng/ml [49]. We collected heart rate and blood pressure (diastolic and systolic) over the course of each session (Baseline, 60 min. [EEfRT], 100 min., 160 min.) to check that the nicotine manipulation was effective.

#### Participants

We used the same participant recruitment strategies and assessment procedures as described in Study 1. Participants were 15 healthy adults (19–30 years old), who reported smoking fewer than five cigarettes per day to minimize effects of tolerance. Exclusion criteria were the same as Study 1. Participants attended a 1-hour orientation session and two 3-hour study sessions. Participants were asked to fast the night before each study session and to abstain from alcohol and substance use (except for their typical caffeine intake) from 24 hours prior to the first study session until after the second study session was completed. At each study session, participants provided urine and breath samples to confirm that they had complied with the study’s guidelines. Similar to study 1, all participants provided written informed consent and all procedures were conducted in accordance with the University of Chicago Institutional Review Board.

#### Analytic approach

All analyses were again mixed models performed in R, using the same packages, coding and random effects models approaches outlined for Study 1.

We conducted parallel analyses of subjective effects for both drugs, but for nicotine we did not expect strong subjective effects in our population and dose. Subjective (DEQ: Feel Drug, and DEQ: Dislike Drug) effects were modeled using a linear mixed effects model (LMM), with fixed effects for Drug, Time (Baseline, 60 min. [EEfRT], 100 min., 160 min.), and their interactions. Instead, for nicotine, our “expected effects” used as manipulation checks were increases in heart rate and blood pressure. These were modeled using a linear mixed effects model (LMM), with fixed effects for Drug, Time (Baseline, 60 min. [EEfRT], 100 min., 160 min.) and their interactions. Omnibus tests were conducted on the models using analysis of variance (ANOVA) Type III based on Satterthwaite’s method.

To test the effect of nicotine on participants’ willingness to exert effort for monetary rewards, we used generalized linear mixed models (GLMMs) with a logit link function for the binomial outcome (choice of easy or hard task). Fixed effects were Nicotine Dose (0, 7mg), Reward Amount for the hard task ($1.24-$4.20), linear and quadratic effects of Probability of receiving a reward (12, 50, 88%), and their interactions. The Probability x Amount interaction represents the expected value of a reward. Additionally, we included fixed effects Trial Number (0–50) and Session (1, 2) to account for fatigue and practice effects over the course of the task or sessions based on previous studies [50]. Omnibus tests were conducted on the model using Type III Wald chi-square tests. Continuous variables of interest were grand-mean centered and converted to Z-scores for all analyses.

## Results

### Study 1 naltrexone

#### Manipulation checks

*Subjective effects of naltrexone*. Compared to placebo, naltrexone significantly increased reports of feeling the drug before the task (at 75 min. post capsule), after the task (at 180 min.), and at the final time point (210 min.); Drug x Time interaction, F(8, 312.67) = 3.82, p = < 0.001. Compared to placebo, both 25mg and 50mg doses of naltrexone also increased reports of disliking a drug effect immediately after the task (at 180 min.) and at the final time point (210 min.); Drug x Time interaction, F(8, 318.19) = 3.79, p < 0.001. Finally, compared to placebo, both doses of naltrexone also increased reports of fatigue immediately after the task (at 180 min.) and at the final time point (210 min.), with 50mg also elevating reports of fatigue immediately before the task (75 min.); Drug x Time interaction, F(8, 318.42) = 3.65, p < 0.001. This suggests the typical effects of naltrexone were present and measurable in our sample at the time of the effort-based decision-making task.

*Cardiovascular effects of naltrexone*. Consistent with our expectations, there was no significant effect of naltrexone on systolic blood pressure F(2, 62.61) = 0.08, p = 0.92, diastolic blood pressure F(2, 31.92) = 1.12, p = 0.34, or heart rate F(2, 31.98) = 0.51, p = 0.60. See S1 Table for full results.

#### EEfRT

Our two hypotheses for naltrexone were assessed using Type III Wald tests for our binomial mixed-effects regression model with choice of the hard task as the dependent variable. Full results are provided in Table 2.

**Table 2:** Naltrexone omnibus effects.

*Predictor*	*X* ^*2*^	*df*	*p*
(Intercept)	13.48	1	**<0.001**
Drug	0.98	2	0.61
Probability	106.31	2	**<0.001**
Amount	156.61	1	**<0.001**
Trials	38.69	1	**<0.001**
Session	4.46	2	0.11
Drug x Probability	2.87	4	0.58
Drug x Amount	0.06	2	0.97
Probability x Amount	61.37	2	**<0.001**
Drug x Trials	1.53	2	0.46
Drug x Probability x Amount	1.31	4	0.86

**Fig 1:**
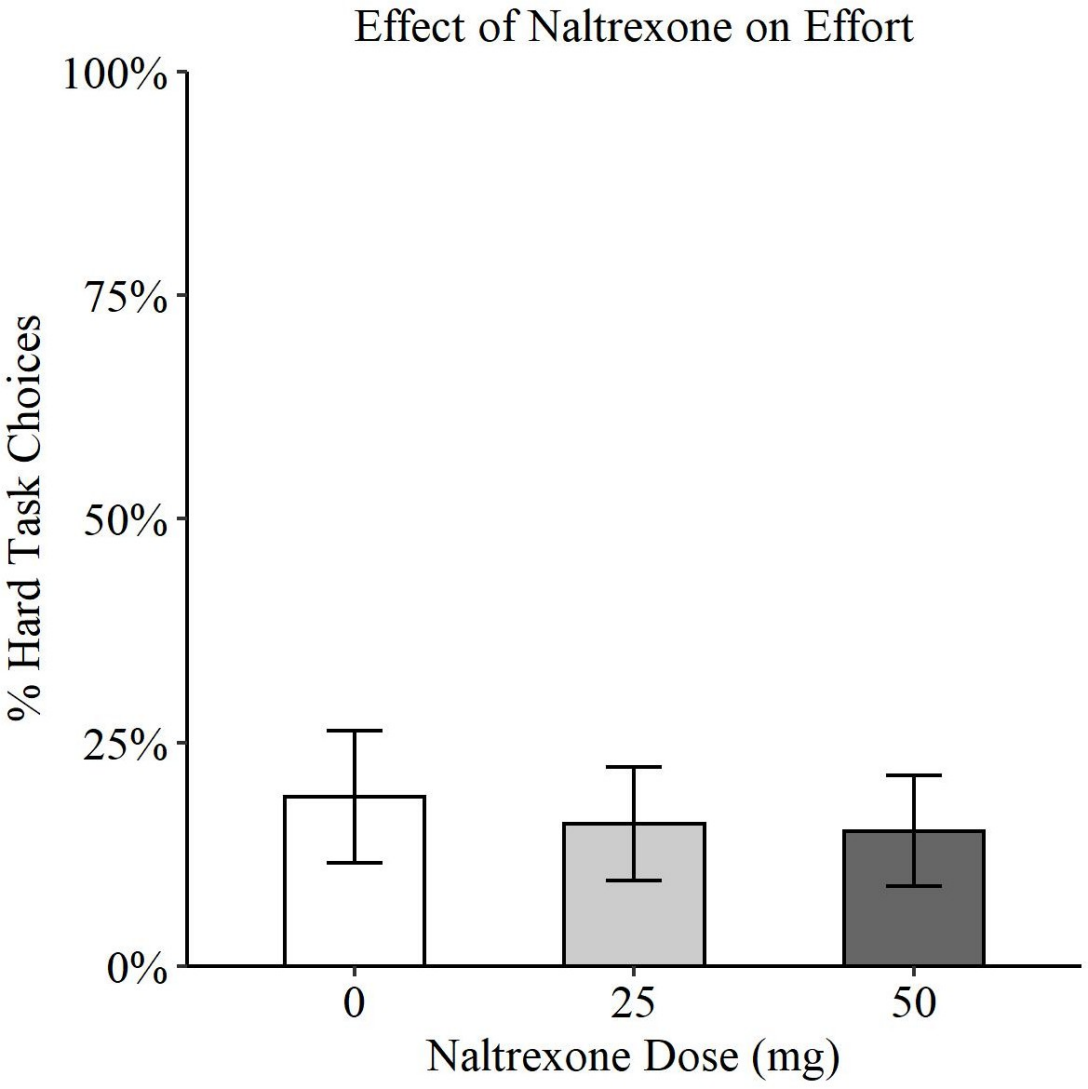
Effect of naltrexone dose (0mg, 25mg, 50mg) on willingness to exert effort in the EEfRT. Estimated marginal means (EMM) are plotted with standard errors (SE) from mixed-effects model (0mg EMM = 0.19, SE = 0.07; naltrexone (25mg) EMM = 0.16, SE = 0.06; naltrexone (50mg) EMM = 0.15, SE = 0.06). No significant effect of naltrexone on choice of hard task (p = 0.61).

The analysis showed no significant effect of naltrexone on choice of the hard task (Wald X^2^(2, N = 34) = 0.98, p = 0.61). See Fig 1.

**Fig 2:**
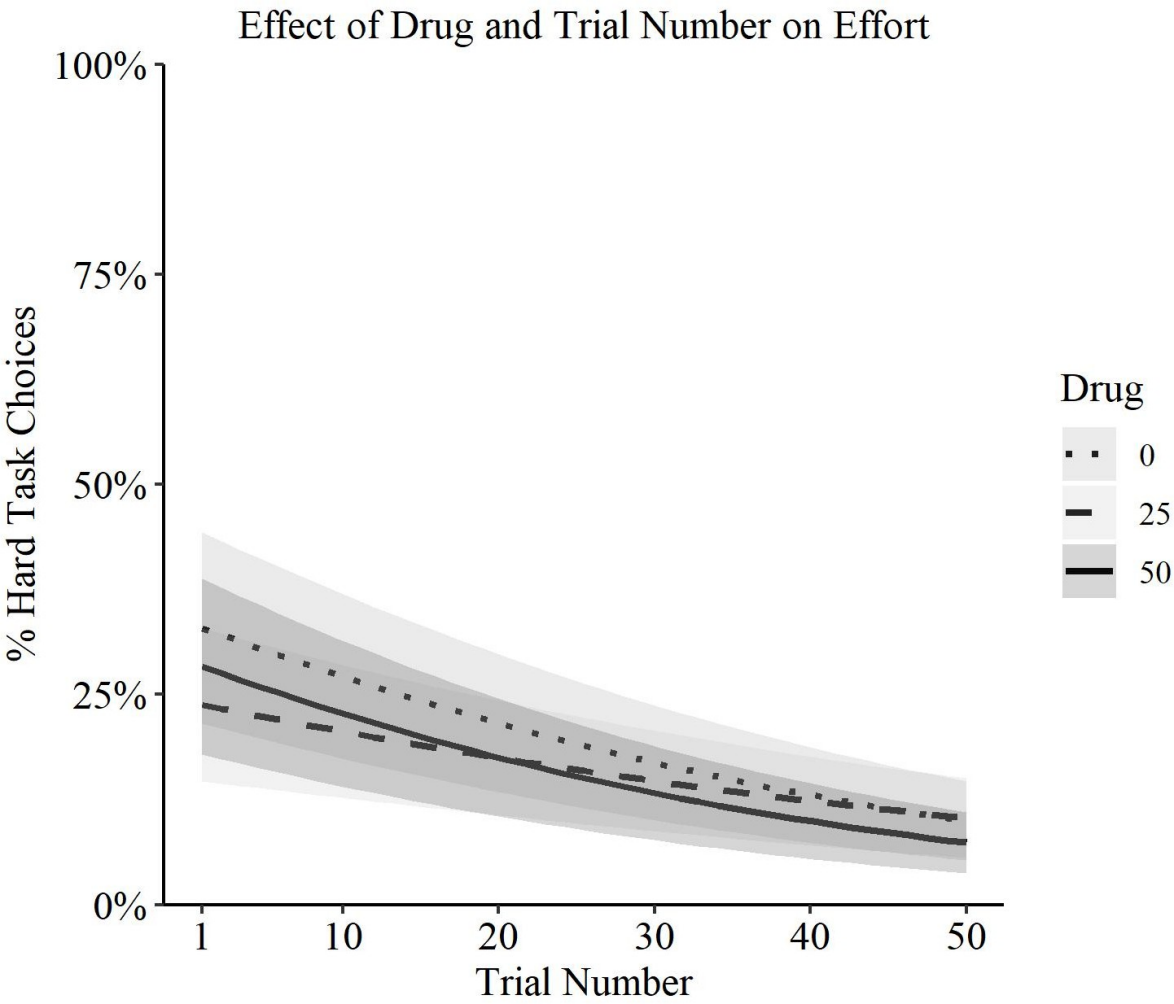
Effect of naltrexone dose (0mg, 25mg, 50mg) on willingness to exert effort across trials. Estimated marginal means are plotted with standard errors shaded from mixed-effects model. No significant interaction between trial number and naltrexone dose (p = 0.46).

Furthermore, although all participants tended to choose the hard task less often in later trials (Wald X^2^(1, N = 34) = 38.69, p < 0.001), naltrexone did not accelerate this tendency, as there was also no significant interaction between trial number and naltrexone dose (Wald X^2^(2, N = 34) = 1.53, p = 0.46; Fig 2).

As expected, as the amount of money that individuals could win for successfully completing the hard task increased, participants chose the hard task more frequently (Wald X^2^(1, N = 34) = 156.61, p < 0.001. Likewise, participants chose the hard task more often when the probability of receiving the monetary reward increased (Wald X^2^(2, N = 34) = 106.31, p < 0.001). Finally, there was a significant interaction between probability and amount such that higher expected values were related to increased choices of the hard task (Wald X^2^(2, N = 34) = 61.37, p < 0.001). Together this all suggests participants attended to the task and their responses were typically sensitive to variations in standard parameters.

### Study 2 nicotine

#### Manipulation checks

*Subjective effects of nicotine*. Consistent with our expectations, nicotine did not significantly increase reports of feeling a drug effect F(1, 70) = 3.54, p = 0.06, or reports of disliking a drug effect F(1, 70) = 0.47, p = 0.50.

*Cardiovascular effects of nicotine*. Cardiovascular effects were consistent with our expectations. Compared to placebo, nicotine significantly increased participants’ systolic blood pressure F(1, 91) = 14.61, p < 0.001, and diastolic blood pressure F(3, 89.98) = 3.13, p < 0.05 before the task (at 60 min.) and after the task (at 100 min.). Nicotine also increased participants’ heart rate after the task (at 100 min.) and at the final time point (at 160 min.); F(3, 89.03) = 4.28, p < 0.01. See S2 Table for full results. Again, this suggests that typical effects of the drug were present and measurable in our sample at the time of the effort-based decision-making task.

#### EEfRT

Similar to our naltrexone analysis, our hypotheses for nicotine were assessed using Type III Wald tests for our binomial mixed-effects regression model with choice for the hard task as the dependent variable. Full results are provided in Table 3.

**Table 3:** Nicotine omnibus effects.

*Predictor*	*X* ^*2*^	*df*	*p*
(Intercept)	0.10	1	0.75
Drug	0.03	1	0.86
Probability	73.61	2	**<0.001**
Amount	32.73	1	**<0.001**
Trials	19.39	1	**<0.001**
Session	3.72	1	0.05
Drug x Probability	7.02	2	**0.03**
Drug x Amount	0.83	1	0.36
Probability x Amount	23.13	2	**<0.001**
Drug x Probability x Amount	0.81	2	0.67

**Fig 3:**
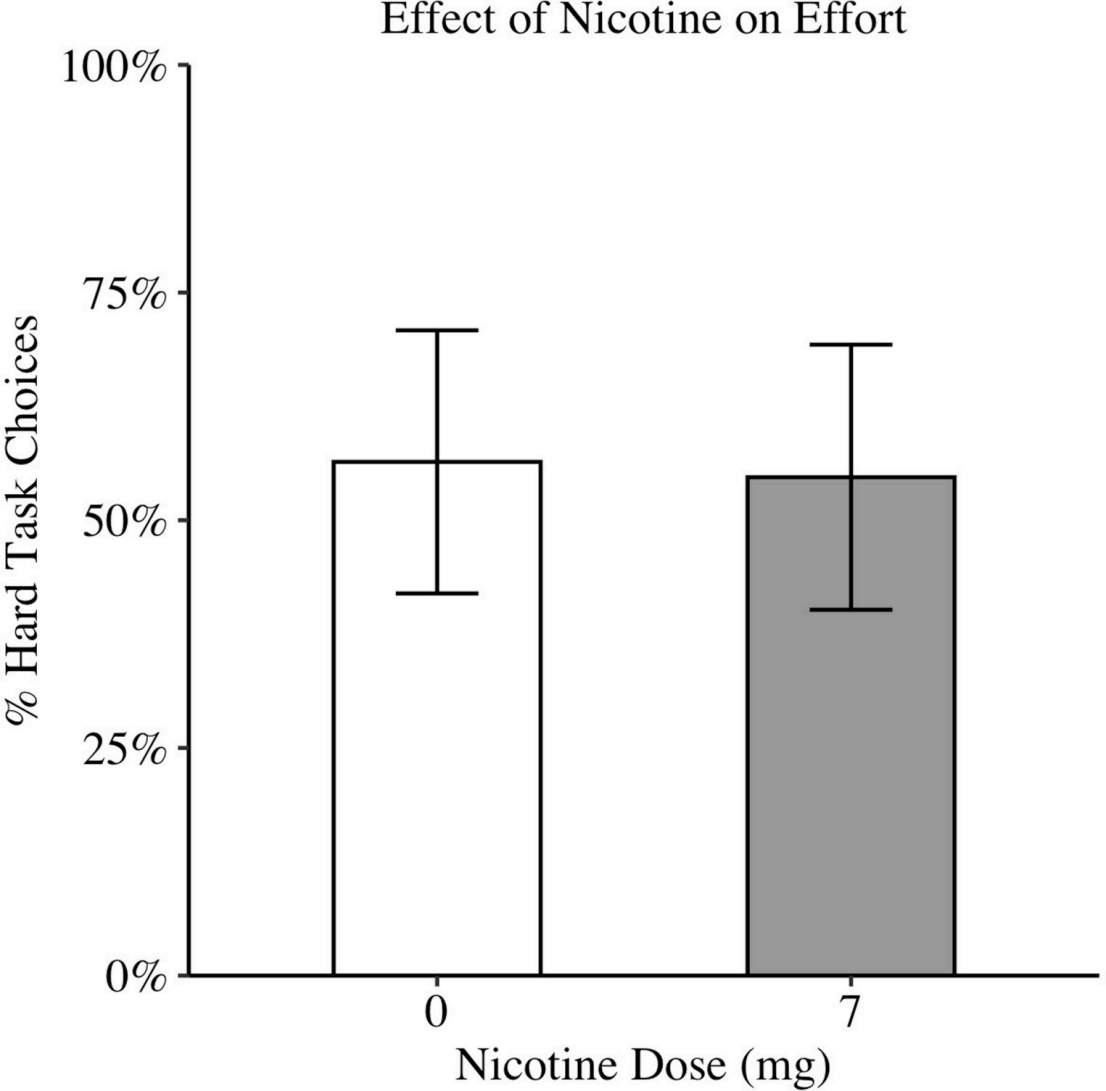
Effect of placebo and nicotine (7mg) on willingness to exert effort on EEfRT. Estimated marginal means are plotted with standard errors from mixed-effects model (Placebo EMM = 0.56, SE = 0.14; Nicotine EMM = 0.55, SE = 0.15). No significant effect of nicotine dose on choice of hard task (p = 0.86).

Nicotine did not significantly affect choice of the hard task (Wald X^2^(1, N = 15) = 0.03, p = 0.86). See Fig 3. Also, contrary to our hypothesis, there were no significant interactions between drug, probability, and amount (Wald X^2^(2, N = 15) = 0.81, p = 0.67).

There was a significant interaction between drug and probability (Wald X^2^(2, N = 15) = 7.02, p = 0.03), suggesting that nicotine had a larger effect on choice of the hard task at medium probability. However, post hoc tests revealed no significant differences between nicotine and placebo on choice of the hard task at low, medium, or high probability. As expected, participants chose the hard task less frequently on later trials (Wald X^2^(1, N = 15) = 19.39, p < 0.001). As the reward amount for successfully completing the hard task increased, participants selection of the hard task also increased (Wald X^2^(1, N = 15) = 32.73, p < 0.001), and selection of the hard task also increased with increasing probability of winning the reward (Wald X^2^(2, N = 15) = 73.61, p < 0.001). Lastly, there was a significant interaction between probability and amount such that higher expected values were related to increased choices of the hard task (Wald X^2^(2, N = 15) = 23.13, p < 0.001). Together this all suggests participants attended to the task and their responses were typically sensitive to variations in standard parameters.

## Discussion

These two studies aimed to determine the acute effects of naltrexone and nicotine on willingness to exert effort for rewards in healthy adults using the EEfRT. Contrary to our hypotheses, neither naltrexone nor nicotine affected individuals’ willingness to exert effort for monetary rewards, even though both drugs produced characteristic effects on other subjective and cardiovascular measures. The results contrast with studies using dopaminergic medications, which affect motivation for effort-based decision making in humans, including on this same task [7–9, 51]. Our results do suggest the participants attended to the task, and that the task sensitively captured a range of reward-related decisions, as we saw typical effects of greater reward magnitudes and higher probability of receiving the reward increasing effort expenditure. They further suggest that the drug doses were adequate, as we saw significant characteristic drug effects on other measures. However, we did not see strong effects of either drug at any level of reward or at any point during the task.

Results from the naltrexone study differed from our expectations based on the prior literature on naltrexone in humans. We expected opioid blockade via naltrexone administration to reduce participants’ willingness to exert effort for rewards, particularly later in the task as participants accumulated experience with the hedonic impact of the rewards. Previous literature showed that naltrexone administration decreased the liking of food rewards and also decreased effort (i.e., lever presses) for drug rewards in rodents [21, 22, 52]. In humans, there were indications that naltrexone would play a similar role, as it slowed reward learning in decision-making tasks and reduced pleasure related to rewarding gambling outcomes [15, 24]. However, we saw neither an overall effect of naltrexone, nor a decrease over trials that could be related to learning about the reduced hedonic value of rewards. The results from our naltrexone study may have differed from our expectations and from previous literature due to the way effort was operationalized. Although previous studies in humans used the same naltrexone dose (50mg), these studies operationalized effort exerted indirectly, as attention allocated during a task or speed of learning. On the other hand, the EEfRT operationalizes effort exertion more directly, as selection for high cost/high rewards, which requires more key presses, over low cost/low rewards. Thus, the effect of naltrexone on willingness to exert effort does not appear to be strong or direct when measured explicitly, although this does not fully rule out more subtle effects that might require a larger sample or different measure to detect.

Regarding nicotine, we expected nicotine to increase participants’ willingness to exert effort for monetary rewards in our pilot study, based on the dual reinforcement theory. In non-human animal models, nicotine amplified the reinforcing value of rewards [28]. However, in humans, nicotine administration has impacted behavioral responding for sensory rewards only in some prior studies [29]. Our results are consistent with the idea that nicotine may only, or may more strongly, impact effort for sensory rewards, as we saw no strong effect on willingness to exert effort for a monetary reward here. Another potential explanation as to why we did not see an effect of nicotine on effort for monetary rewards may be due to sample differences. Some studies that did suggest nicotine enhancement of responsiveness for financial rewards only found this effect among individuals who were heavy smokers (i.e., reported smoking 15 or more cigarettes per day) [32]. However, in our sample, we only included light-smoking non-dependent individuals who reported smoking fewer than five cigarettes per day. Other studies that have included a similar sample to ours (i.e., reported smoking less than five cigarettes per day) also found no reinforcing effect of nicotine on money [35]. It is possible that the dual reinforcing effects of nicotine are stronger or more generalized in individuals who smoke more heavily, or that the effects on monetary rewards seen in these prior studies represented withdrawal relief rather than true enhancement.

These studies had several limitations. For both studies, the drug dosages were fixed, not based on individual bodyweight and body mass composition. However, the doses used for naltrexone are typical of those used clinically in treatment, while the dose of nicotine is a typical dose used for nicotine replacement therapy, suggesting these doses and results have real-world relevance in terms of the potential for effort-related side effects of these drugs. Second, due to the limitations of the dose response design in the parent study [41], we only tested the effects of an opioid antagonist and not an agonist. Future studies examining opioid agonists in relation to effort-based decision-making may provide additional insight into the role of the opioid system in this aspect of reward functioning. Third, in our naltrexone study, willingness to exert effort was lower than expected compared to other studies on the EEfRT [37], including our pilot study on nicotine, which may be related to unmeasured differences in the study settings or samples. Future studies examining the relationship between naltrexone and willingness to exert effort should consider replicating the laboratory conditions used in other EEfRT studies. Fourth, we did not collect baseline measures of effort. Previous studies in laboratory animals have suggested that baseline differences may determine the degree to which pharmacological manipulations change effort for rewards, including for nicotine [31]. We also did not directly contrast sensory and non-sensory rewards. Future studies of pharmacological manipulations should consider simultaneously investigating impacts on sensory rewards vs. non-sensory rewards to investigate this intriguing difference observed in the nicotine literature. Fifth, we did not collect measures of other variables that may help further understand effort-based decision-making, such as acute motivational state and trait characteristics. Future studies should consider including measures of these variables. Finally, our sample sizes were small, although consistent with the sample sizes needed to detect the effects of other pharmacological manipulations on this measure (e.g., 7,41), and adequate to demonstrate typical effects of the drugs on other measures. For naltrexone, a small observed effect size (d = 0.26) between placebo and 25mg and between placebo and 50mg (d = 0.24) indicate a study with N = 139 would be necessary to detect a significant effect of naltrexone dose. For nicotine, the very small effect size (d = 0.09) between placebo and nicotine in our pilot study suggests a study with N = 876 would be needed to detect a significant effect of nicotine. Thus, it seems unlikely that these effects would be strongly meaningful even if they might be detectable with a larger sample. Nonetheless, since our nicotine was a pilot study, it provides preliminary information, and it may be beneficial to conduct additional studies on nicotine and willingness to exert effort.

Overall, our analyses suggest neither naltrexone nor nicotine strongly impact effort exerted for monetary rewards. Our findings have some implications for the use of naltrexone for treatment of opioid use disorder. Although there has been some concern that naltrexone treatment of opioid use disorder could increase anhedonia [53], our results suggest this is not a major concern and are consistent with survey research with patients receiving the drug as treatment [53, 54]. Our nicotine findings also add to our understanding of the mechanisms by which nicotine may become addictive and may inform treatment interventions. Previous literature suggests nicotine primarily impacts the reinforcing effects of sensory rewards (i.e., audio, olfactory, taste, visual stimuli) and not non-sensory rewards (i.e., money) [29], which our findings suggested. Ultimately, this may be helpful for designing interventions to assist individuals who are attempting to quit smoking, as lapses may be more common in the context of sensory rewarding activities, while monetary rewards may be more consistently valued. This possibility is consistent with the efficacy of interventions such as contingency management, which offers monetary rewards to motivate cessation. In summary, despite studies in laboratory animals showing that opioidergic and nicotinergic systems influence effort-based decision-making, two commonly used substances in humans that affect these systems did not strongly affect willingness to exert effort for rewards at doses typically used in humans.

## Supporting information

S1 Table Naltrexone manipulation checks. Omnibus Effects for Feel Drug on the Drug Effectiveness Questionnaire (DEQ), Dislike Drug on the DEQ, Fatigue on the Profile of Mood States, Systolic Blood Pressure, Diastolic Blood Pressure, and Heart Rate.
(DOCX)

S2 Table Nicotine manipulation checks. Omnibus Effects for Feel Drug on the Drug Effectiveness Questionnaire (DEQ), Dislike Drug on the DEQ, Systolic Blood Pressure, Diastolic Blood Pressure, and Heart Rate.
(DOCX)
