# Supplementary material for: Pharmacological investigations of effort-based decision-making in humans: Naltrexone and nicotine
Source: PLoS One. 2022 Oct 5;17(10):e0275027. doi: 10.1371/journal.pone.0275027 (PMC9534411; doi:10.1371/journal.pone.0275027)
Supplement: S1 Table — Omnibus Effects for Feel Drug on the Drug Effectiveness Questionnaire (DEQ), Dislike Drug on the DEQ, Fatigue on the Profile of Mood States, Systolic Blood Pressure, Diastolic Blood Pressure, and Heart Rate. (DOCX) [file pone.0275027.s001.docx]

**S1 Table.** **Naltrexone Manipulation Checks**. Omnibus Effects for Feel Drug on the Drug Effectiveness Questionnaire (DEQ), Dislike Drug on the DEQ, Fatigue on the Profile of Mood States, Systolic Blood Pressure, Diastolic Blood Pressure, and Heart Rate.

|  | **Feel Drug** | | | | |
| --- | --- | --- | --- | --- | --- |
| *Predictor* | *df* | *SS* | *MS* | *F* | *p* |
| Time | 4 | 6401.0 | 1600.3 | 13.45 | **<0.001** |
| Drug | 2 | 830.1 | 415.1 | 3.49 | **<0.05** |
| Time x Drug | 8 | 3636.5 | 454.6 | 3.82 | **<0.001** |
|  |  |  | | | |

|  | **Dislike Drug** | | | | |
| --- | --- | --- | --- | --- | --- |
| *Predictor* | *df* | *SS* | *MS* | *F* | *p* |
| Time | 4 | 10897.3 | 2724.3 | 9.69 | **<0.001** |
| Drug | 2 | 2407.4 | 1203.7 | 4.28 | **<0.05** |
| Time x Drug | 8 | 8526.9 | 1065.9 | 3.79 | **<0.001** |

|  | **Fatigue** | | | | |
| --- | --- | --- | --- | --- | --- |
| *Predictor* | *df* | *SS* | *MS* | *F* | *p* |
| Time | 4 | 164.9 | 41.2 | 11.07 | **<0.001** |
| Drug | 2 | 27.5 | 13.7 | 3.69 | **<0.05** |
| Time x Drug | 8 | 108.8 | 13.6 | 3.65 | **<0.001** |

|  | **Systolic Blood Pressure** | | | | |
| --- | --- | --- | --- | --- | --- |
| *Predictor* | *df* | *SS* | *MS* | *F* | *p* |
| Time | 4 | 1965.1 | 491.3 | 10.6 | **<0.001** |
| Drug | 2 | 7.6 | 3.8 | 0.08 | 0.92 |
| Time x Drug | 8 | 203.8 | 25.5 | 0.55 | **0.82** |

|  | **Diastolic Blood Pressure** | | | | |
| --- | --- | --- | --- | --- | --- |
| *Predictor* | *df* | *SS* | *MS* | *F* | *p* |
| Time | 4 | 487.6 | 121.9 | 3.93 | **<0.01** |
| Drug | 2 | 69.6 | 34.8 | 1.12 | 0.34 |
| Time x Drug | 8 | 399.0 | 49.9 | 1.61 | **0.12** |

|  | **Heart Rate** | | | | |
| --- | --- | --- | --- | --- | --- |
| *Predictor* | *df* | *SS* | *MS* | *F* | *p* |
| Time | 4 | 3510.3 | 877.6 | 29.73 | **<0.001** |
| Drug | 2 | 30.3 | 15.1 | 0.51 | 0.60 |
| Time x Drug | 8 | 216.4 | 27.1 | 0.92 | **0.50** |
