# Supplementary material for: Pharmacological investigations of effort-based decision-making in humans: Naltrexone and nicotine
Source: PLoS One. 2022 Oct 5;17(10):e0275027. doi: 10.1371/journal.pone.0275027 (PMC9534411; doi:10.1371/journal.pone.0275027)
Supplement: S2 Table — Omnibus Effects for Feel Drug on the Drug Effectiveness Questionnaire (DEQ), Dislike Drug on the DEQ, Systolic Blood Pressure, Diastolic Blood Pressure, and Heart Rate. (DOCX) [file pone.0275027.s002.docx]

**S2 Table.** **Nicotine Manipulation Checks**. Omnibus Effects for Feel Drug on the Drug Effectiveness Questionnaire (DEQ), Dislike Drug on the DEQ, Systolic Blood Pressure, Diastolic Blood Pressure, and Heart Rate.

|  | **Feel Drug** | | | | |
| --- | --- | --- | --- | --- | --- |
| *Predictor* | *df* | *SS* | *MS* | *F* | *p* |
| Time | 2 | 1171.4 | 585.7 | 2.38 | 0.10 |
| Drug | 1 | 871.1 | 871.1 | 3.54 | 0.06 |
| Time x Drug | 2 | 31.5 | 15.7 | 0.06 | 0.93 |

|  | **Dislike Drug** | | | | |
| --- | --- | --- | --- | --- | --- |
| *Predictor* | *df* | *SS* | *MS* | *F* | *p* |
| Time | 2 | 316.7 | 158.3 | 0.63 | 0.54 |
| Drug | 1 | 117.9 | 117.9 | 0.47 | 0.50 |
| Time x Drug | 2 | 497.5 | 248.7 | 0.99 | 0.38 |

|  | **Systolic Blood Pressure** | | | | |
| --- | --- | --- | --- | --- | --- |
| *Predictor* | *df* | *SS* | *MS* | *F* | *p* |
| Time | 3 | 78.0 | 26.0 | 0.60 | 0.62 |
| Drug | 1 | 636.5 | 636.5 | 14.61 | **<0.001** |
| Time x Drug | 3 | 294.4 | 98.1 | 2.25 | 0.09 |

|  | **Diastolic Blood Pressure** | | | | |
| --- | --- | --- | --- | --- | --- |
| *Predictor* | *df* | *SS* | *MS* | *F* | *p* |
| Time | 3 | 187.1 | 62.4 | 1.55 | 0.21 |
| Drug | 1 | 271.7 | 271.7 | 6.76 | **<0.05** |
| Time x Drug | 3 | 377.1 | 125.7 | 3.13 | **<0.05** |

|  | **Heart Rate** | | | | |
| --- | --- | --- | --- | --- | --- |
| *Predictor* | *df* | *SS* | *MS* | *F* | *p* |
| Time | 3 | 160.7 | 53.6 | 1.56 | 0.20 |
| Drug | 1 | 317.3 | 317.3 | 9.23 | **<0.01** |
| Time x Drug | 3 | 441.2 | 147.1 | 4.28 | **<0.01** |
